# Seroprevalence of *Toxoplasma gondii* and Associated Risk Factors among Pregnant Women in Algeria

**DOI:** 10.4269/ajtmh.23-0187

**Published:** 2024-04-30

**Authors:** Soumia Sebaa, Jerzy M. Behnke, Amina Labed, Marawan A. Abu-Madi

**Affiliations:** ^1^ Laboratory of Exploration and Valorization of Steppic Ecosystems, Faculty SNV, University of Ziane Achour, Djelfa, Algeria;; ^2^ School of Life Sciences, University of Nottingham, University Park, Nottingham, United Kingdom;; ^3^ Department of Ecology and Environment, University Batna 2, Batna, Algeria;; ^4^ Department of Biomedical Science, Biomedical Research Centre, College of Health Sciences, Qatar University, Doha, Qatar

## Abstract

*Toxoplasma gondii* is an obligate intracellular protozoan parasite affecting all warm-blooded vertebrates, including humans. Infections in humans can lead to severe clinical manifestations in pregnant women and immunocompromised patients. The aim of the present study was to assess seroprevalence of *T. gondii* infection and to identify the associated risk factors among pregnant women from southern Algeria. A cross-sectional study was conducted from March 2021 to May 2022 among 1,345 pregnant women. A structured questionnaire was used to obtain information on risk factors associated with infection. Participants were screened for anti *T. gondii* IgG and IgM antibodies using the enzyme-linked fluorescent assay. The overall IgG and IgM seroprevalence was 13.6% and 0.89%, respectively. A significant association was found between seroprevalence of anti *T. gondii* IgG and history of spontaneous abortion (*P* = 0.016). Moreover, an increasing trend of seroprevalence was detected in the autumnal season (*P* = 0.030), and eating raw or undercooked meat was found to be significantly associated with anti–*T. gondii* IgM seropositivity (*P* = 0.002). Multivariate analysis showed that women who had experienced spontaneous abortion, regularly ate undercooked meat, and used bottled water in summer were more likely to contract infection with *T. gondii*. The majority (86.4%) of the studied pregnant women were serologically negative for toxoplasmosis and hence were susceptible to primary acute infection during pregnancy and possible fetal anomalies. Therefore, health education and awareness of the disease and its transmission to women, especially during pregnancy, is imperative.

## INTRODUCTION

Toxoplasmosis is a common worldwide parasitic zoonoses, caused by an obligate intracellular protozoan parasite, *Toxoplasma gondii*.^1^ Although most infections are asymptomatic, infection can have severe consequences if pregnant women become infected. *Toxoplasma gondii* is capable of crossing the transplacental barrier from a primo-infected pregnant woman to her fetus,^2^ leading to congenital toxoplasmosis, although the risk of fetal transmission depends on the exact timing of exposure during pregnancy. Consequently, depending on the trimester of pregnancy, infection with *T. gondii* can cause not only miscarriage or stillbirth but also serious and progressive visual, hearing, motor, and cognitive complications for the child.^3^ Therefore, early diagnosis of toxoplasmosis crucial to enable treatment in sufficient time to minimize transplacental transmission. In the majority of cases, toxoplasmosis is an asymptomatic disease in adults, but it can have devastating consequences in immunocompromised patients and nonimmunized pregnant women.^4^^,^^5^ *Toxoplasma gondii* is commonly transmitted to humans by accidental ingestion of the oocyst stages of the parasite in water or food, in soil contaminated with feces from infected cats, or by eating raw or undercooked meat containing tachyzoites or bradyzoites.^6^^,^^7^

Infection is usually asymptomatic, thus it is most often detected by serological testing in pregnant women, although clinical analysis can contribute in subjects showing acute signs of infection. *Toxoplasma*-specific antibodies IgM and IgG can be detected in the serum of pregnant women within 1 to 2 weeks of exposure to infection.^8^ In patients with *Toxoplasma* infection, it is important to establish whether they have an acute or chronic infection. Additional screening through the avidity test of *T. gondii*–specific IgG antibodies^9^ and polymerase chain reaction analysis of the amniotic fluid are usually used for greater certainty.^10^ A positive diagnosis of acute infection in a pregnant woman indicates a high risk of potential transmission of the parasite to the fetus, and this should trigger treatment to prevent vertical transmission.^11^

Prevalence of toxoplasmosis is known to vary widely between countries.^12^ In Algeria, seroprevalence of toxoplasmosis during pregnancy has been poorly reported.^10^^,^^13^^,^^14^ The overall seroprevalence of *T. gondii* infection ranges from 25% to 52.6%, with seroprevalence of recent *Toxoplasma*-specific IgM antibodies ranging from 1.1% to 1.6%^13^^,^^14^ and prevalence of acute infection for both IgG and IgM antibodies ranging from 4.8% to 43.8%.^10^^,^^14^ However, there is scarcity of information on the risk factors for contracting *T. gondii* infection among pregnant women in Algeria, and there are no published reports on *T. gondii* infection in Laghouat province. This study therefore sought to determine seroprevalence of *T. gondii* and to identify the potential risk factors associated with *T. gondii* infection among pregnant women in Laghouat province, southern Algeria.

## MATERIALS AND METHODS

### Study area.

This study was carried out in the province of Laghouat, situated in the central south of Algeria, 400 km to the south of the capital Algiers, between latitude 33° 48′ north and longitude 02° 53′ east. It is characterized by agro-pastoral activity and covers ∼25,052 km^2^, with an estimated population of 716,219 inhabitants in 2020. In total, 12,820 pregnant women attend the maternity clinic per year with a birth rate estimated at 98.8%, and a predicted 10-year population growth rate of 3.8% (unpublished observations, monograph of the wilaya of Laghouat). Daily temperatures average −5°C in winter months and >40°C in summer, and the average annual rainfall is 151.21 mm (Unpublished data, Bouchetata, 2018). Agricultural activities such as livestock husbandry and breeding (sheep, goats, cattle, horses, camels, and chicken) are the main economic activities in the rural population, and water is available through boreholes and taps supplied by wells or public water delivery systems (Unpublished data, Directorate of Agricultural Services, 2016).

### Selection of subjects and study design.

A cross-sectional survey was conducted among all apparently healthy pregnant women who agreed to participate in the study from March 2021 to May 2022 and who had been referred to three private laboratories conducting medical analyses in Laghouat province. In Algeria, serological screening of pregnant women for *T. gondii* infection is routinely requested by gynecologists. Data were collected by laboratory personnel using a structured questionnaire constructed in French and translated into Arabic, the local language. Pregnant women (*N* = 1,345) were interviewed on the day of blood sample collection to acquire data about the potential risk factors for toxoplasmosis including age, symptoms, residential area, stage of pregnancy, any previous experience of abortion, contact with cats, water consumption, consumption of undercooked meat, and exposure to soil. Questionnaires in Arabic guided the face-to-face interviews conducted by the investigators, who briefed the participants about the study objectives. Questions were answered orally by the interviewees, and the answers were recorded by the investigators for subsequent conversion into digital format and addition to the database used for statistical analysis. Incomplete patient records and, to avoid bias in the results, cases of women who returned for follow-up to earlier examination were excluded from this study because the data for analysis were restricted to cases of pregnant women visiting the laboratory for the first time.

### Sample collection and ethical approval.

Ethical clearance for the study was obtained from the Faculty of Science of Nature and Life, Djelfa University, Algeria (Ref:AT04/E.V.E.S.). The study objectives and procedures for sample collection were explained to participants, and informed written consent was obtained. Approximately 5 mL of venous blood were collected from each pregnant woman in a tube coated in EDTA. Each tube was centrifuged at 3,000 × *g* for 5 minutes, and the collected plasma was tested directly.

### Serological testing.

Serological diagnosis of toxoplasmosis was performed assessing presence of both *Toxoplasma*-specific IgG and IgM antibodies in participants’ sera by the three private medical laboratories in Laghouat province, all using identical techniques. The sera were analyzed using a standard enzyme-linked fluorescent assay using the Vitek Immuno-Diagnostic Assay System (VIDAS Toxo IgG II and IgM, bio Mérieux-Marcy-l’Etoile, France) run in a VIDAS fully automated immunoanalyzer. The system was calibrated and standard operating procedures were implemented following the manufacturer’s instructions. A titer of IgG anti-*Toxoplasma* antibody ≥8 IU/mL was considered positive in this study, equivocal results were defined as IgG values ranging from 4 to 8 IU/mL, and negative results were defined as IgG values of <4 IU/mL. Samples were considered reactive for IgM when the antibody concentration was greater than or equal to an index of 0.65, nonreactive when the concentration was <0.55 index, and undetermined when the concentration was between 0.55 and 0.65 index.

## STATISTICAL ANALYSES

For data subsets, prevalence values (percentage of subjects with positive IgG or IgM titers) are given with 95% confidence limits (CL_95_) in the text and 95% CIs in figures, and calculated in bespoke software based on the tables of Rohlf and Sokal.^15^ Odds ratios + 95% CLs were calculated for levels within each factor, using one level as the reference point in each case.

Prevalences were analyzed using maximum likelihood techniques based on log linear analysis of contingency tables in the software package IBM SPSS, v. 28.0 (IBM Corp., Armonk, NY). This approach is based on categorical values of factors of interest, which are used not only to fit hierarchical log-linear models to multidimensional cross-tabulations using an iterative proportional-fitting algorithm but also to detect associations between factors, one of which is the presence or absence of IgG or IgM. We first fitted models with presence/absence of IgG positivity and each of the explanatory factors in turn. Explanatory factors included age class (eight levels corresponding to age class 1 [19.0–21.9], age class 2 [22.0–24.9], age class 3 [25.0–27.9 years old], age class 4 [28.0–31.9 years old], age class 5 [32.0–34.9 years old], age class 6 [35.0–37.9 years old], age class 7 [38.0–40.9 years old], and age class 8 [41.0–44.9]), year (two levels, 2021 and 2022), trimester of pregnancy (three levels, first, second, and third trimester), history of abortion (two levels, experienced spontaneous abortion or not), season (four levels, spring, summer, autumn, and winter), location (two levels, rural or urban), use of water (bottled or tap), possession of a pet cat (two levels, yes or no), consumption of meat (well-cooked or poorly cooked), and possession of a garden (two levels, yes or no).

Next, we fitted multifactorial models in three stages as described previously^16^ because of the number of factors involved. First, we fitting a model with intrinsic explanatory factors, then one with extrinsic factors, and finally we fitted a model with all the factors that had shown *P*-values of <0.3 in the first two stages. By employing the backward selection procedure in SPSS, we simplified each model until only significant terms remained. For each level of analysis in turn, beginning with the most complex model, involving all possible main effects and interactions, those combinations that did not contribute significantly to explaining variation in the data were eliminated in a stepwise fashion beginning with the highest level interaction (backward selection procedure). A minimum sufficient model was then obtained, for which the likelihood ratio of χ^2^ was not significant, indicating that the model was sufficient in explaining the data. The importance of each term in interactions involving presence or absence of IgG or IgM in the final model was assessed by the probability that its exclusion would alter the model significantly and these values are given in the text, assessed by a likelihood ratio test between models with and without each term of interest.

## RESULTS

### Sociodemographic characteristics.

A total of 1,345 pregnant women aged between 19 and 44 years, with a mean age of 29.37 were enrolled during the study period. Approximately one-quarter of the study population were in the age range of 28–31 years (24.6%). One hundred twenty-nine (10%) of the participants had a history of spontaneous abortion. Of all participants, 753 (55.9%) were urban and 592 (44.1%) were rural residents. With regard to the gestational age, it was found that 66.5% of the women were in the first trimester of pregnancy, 27.3% were in the second trimester and 6.2% were in the third trimester.

### Seroprevalence of *T. gondii* infection.

The prevalence of specific anti *T. gondii* IgG was 13.6% (95% CI: 11.77–15.44), and that of IgM considerably lower at 0.89% (95% CI: 0.461–1.558). Prevalence by levels within the factors, together with odds ratios and comparison of odds ratios to the first listed level within each factor, are summarized in Table 1 (IgG) and Table 2 (IgM).

**Table 1:** Percentage of subjects showing positive anti *Toxoplasma gondii* IgG values in data subsets corresponding to the risk factors that were implemented in the study and odds ratios comparing each level to the first listed within each respective factor

Level	*n*	%	CL_95_	OR	CL_95_	*z*	*P*-Value
Intrinsic factors							
Age class							
1	222	12.6	9.83–16.00	–	–	–	–
2	157	15.3	9.89–22.53	1.250	0.694–2.251	0.744	0.228
3	217	13.4	10.53–16.79	1.069	0.613–1.865	0.234	0.407
4	331	13.9	10.44–18.17	1.118	0.676–1.851	0.435	0.331
5	127	11.0	6.84–16.93	0.858	0.434–1.698	−0.439	0.330
6	125	13.6	9.02–19.73	1.091	0.571–2.083	0.263	0.396
7	120	12.5	8.22–18.34	0.990	0.506–1.935	−0.030	0.488
8	46	21.7	10.56–39.07	1.925	0.861–4.304	1.594	0.055
Trimester of pregnancy							
1	895	12.9	10.30–15.89	–	–	–	–
2	367	15.0	11.24–19.65	1.196	0.845–1.692	1.009	0.156
3	83	15.7	8.11–26.99	1.260	0.675–2.350	0.726	0.234
History of abortion							
None	1,216	12.8	10.95–14.71	–	–	–	–
Yes	129	20.9	15.11–27.93	1.799	1.140–2.839	2.522	0.016*
Extrinsic factors							
Year							
2021	697	13.8	11.44−16.47	–	–	–	–
2022	648	13.4	11.20−15.99	0.971	0.711–1.327	−0.186	0.426
Season							
Spring	449	11.4	7.71–16.19	–	–	–	–
Summer	128	12.5	8.10–18.54	1.115	0.612–2.030	0.355	0.361
Autumn	385	15.8	11.90–20.69	1.469	0.985–2.191	1.886	0.030*
Winter	383	14.4	10.59–19.05	1.309	0.870–1.968	1.292	0.098
Location							
Urban	753	14.1	11.63–16.92	–	–	–	–
Rural	592	13.0	10.91–15.43	0.913	0.666–1.251	−0.568	0.285
Lifestyle and behavioral factors							
Use of water							
Bottled	742	13.9	11.47–16.68	–	–	–	–
Tap	603	13.3	11.12–15.73	0.949	0.693–1.299	−0.327	0.372
Possession of domestic cat							
No	799	12.9	10.49–15.73	–	–	–	–
Yes	546	14.7	12.49–17.07	1.160	0.847–1.589	0.924	0.177
Consumption of meat							
Well–cooked	1,014	13.6	11.50–15.72	–	–	–	–
Poorly cooked	331	13.6	10.19–17.85	0.999	0.695–1.434	−0.007	0.497
Garden							
No	883	12.8	10.27–15.81	–	–	–	–
Yes	462	15.2	10.92–20.55	1.217	0.882–1.679	1.195	0.116

ORs = odds ratios. ORs are calculated in relation to the first listed level under each factor.

* Statistically significant.

**Table 2:** Percentage of subjects showing positive anti *Toxoplasm gondii* IgM values in data subsets corresponding to the risk factors that were implemented in the study and odds ratios comparing each level to the first listed within each respective factor

Level	*N*	%	CL_95_	OR	CL_95_	*z*	*P*-Value
Intrinsic factors							
Age class							
1	222	1.35	0.615–2.910	–	–	–	–
2	157	1.27	0.218–5.083	0.942	0.156–5.704	−0.065	0.474
3	217	1.84	0.945–3.537	1.371	0.303–6.198	0.410	0.341
4	331	0.00	0.000–1.239	0	–	–	–
5	127	0.79	0.116–3.731	0.579	0.060–5.629	−0.471	0.319
6	125	0.80	0.120–3.718	0.589	0.061–5.721	−0.457	0.324
7	120	0.83	0.130–3.689	0.613	0.063–5.963	−0.421	0.337
8	46	0.00	0.000–10.418	0	–	–	–
Trimester of pregnancy							
1	895	1.01	0.433–2.248	–	–	–	–
2	367	0.54	0.151–2.297	0.539	0.116–2.509	−0.787	0.216
3	83	1.20	0.088–8.147	1.201	0.150–9.594	0.172	0.432
History of abortion							
None	1,216	0.90	0.452–1.619	–	–	–	–
Yes	129	0.78	0.113–3.745	0.856	0.110–6.683	−0.148	0.441
Extrinsic factors							
Year							
2021	697	1.00	0.488–2.040	–	–	–	–
2022	648	0.77	0.385–1.641	0.766	0.242–2.427	−0.452	0.326
Season							
Spring	449	1.34	0.398–3.914	–	–	–	–
Summer	128	1.56	0.400–5.026	1.172	0.234–5.878	0.193	0.424
Autumn	385	0.78	0.206–2.769	0.580	0.144–2.334	−0.767	0.222
Winter	383	0.26	0.069–1.878	0.193	0.023–1.613	−1.519	0.064
Location							
Urban	753	0.66	0.312–1.579	–	–	–	–
Rural	592	1.18	0.648–2.156	1.790	0.565–5.669	0.990	0.161
Lifestyle and behavioral factors							
Use of water							
Bottled	742	0.81	0.382–1.789	–	–	–	–
Tap	603	1.00	0.509–1.930	1.233	0.396–3.842	0.361	0.359
Possession of domestic cat							
No	799	0.88	0.400–1.950	–	–	–	–
Yes	546	0.92	0.483–1.754	1.046	0.330–3.312	0.076–0.470	–
Consumption of meat							
Well-cooked	1,014	0.39	0.107–1.010	–	–	–	–
Poorly cooked	331	2.42	1.183–4.822	6.254	1.871–20.905	2.977	0.001*
Garden							
No	883	0.79	0.343–1.904	–	–	–	–
Yes	462	1.08	0.251–3.598	1.369	0.432–4.338	0.534	0.297

ORs = odds ratios. ORs are calculated in relation to the first listed level under each factor.

* Statistically significant.

### Risk factors associated with IgG anti *T. gondii* infection.

The prevalence of IgG positive samples was remarkably consistent across eight age classes (χ^2^_7_ = 3.73, *P =* 0.81) and the trimesters of pregnancy (χ^2^_2_ = 1.31, *P =* 0.52). As for the obstetric variables, the pregnant women who had a spontaneous abortion history showed a significantly higher seroprevalence of *T. gondii* infection (20.9% versus 12.8%; χ^2^_1_ = 5.820, *P =* 0.016). Although there was no overall significant effect of season of the year (χ^2^_3_ = 3.92, *P =* 0.27), based on comparison of odds ratios, prevalence was significantly, but not substantially, higher in autumn compared with spring (Table 1). There was no significant difference in prevalence between the 2 years of the study (χ^2^_1_ = 0.034, *P =* 0.85), whether subjects lived in rural or urban areas (χ^2^_1_ = 0.32, *P =* 0.57), relied on bottled or tap water (χ^2^_1_ = 0.11, *P =* 0.74), had pet cats or not (χ^2^_1_ = 0.85, *P =* 0.36), consumed well or poorly cooked meat (χ^2^_1_ <0.001, *P =* 0.99), or had access to gardens or not (*χ*^2^_1_ = 1.41, *P =* 0.24).

Multifactorial analysis of intrinsic factors (age class, stage of trimester and history of abortion plus presence/absence of IgG positivity) revealed that only history of abortion was significant. Multifactorial analysis of extrinsic and behavioral factors did not reveal any significant interactions, and a final model in which we included only those factors for which *P*-values in the first two stages were ≤0.3 (garden, season, and history of abortion) found only one significant main effect (history of abortion, χ^2^_1_ = 5.820, *P =* 0.016) but no significant interactions.

### Risk factors associated with IgM anti *T. gondii* infection.

IgM positive samples were rare, there being only 12 positive samples in the entire dataset. Perhaps not surprisingly, there was no significant main effect of most factors (age class, χ^2^_7_ = 9.17, *P =* 0.24; trimesters of pregnancy, χ^2^_2_ = 0.79, *P =* 0.67; history of abortion, χ^2^_1_ = 0.023, *P =* 0.879; years, χ^2^_1_ = 0.21, *P =* 0.65; seasons, χ^2^_3_ = 3.85, *P =* 0.28; location, χ^2^_1_ = 1.00, *P =* 0.32; water consumption, χ^2^_1_ = 0.13, *P =* 0.72; possession of a cat, χ^2^_1_ = 0.01, *P =* 0.94; and use of a garden, χ^2^_1_ = 0.28, *P =* 0.60). However, there was a significantly higher prevalence of IgM positive samples among those who did not cook their meat thoroughly compared with those who did (χ^2^_1_ = 9.521, *P =* 0.002). Prevalence was 6 times higher in the former group (Table 2, odds ratio = 6.254).

**Figure 1.**
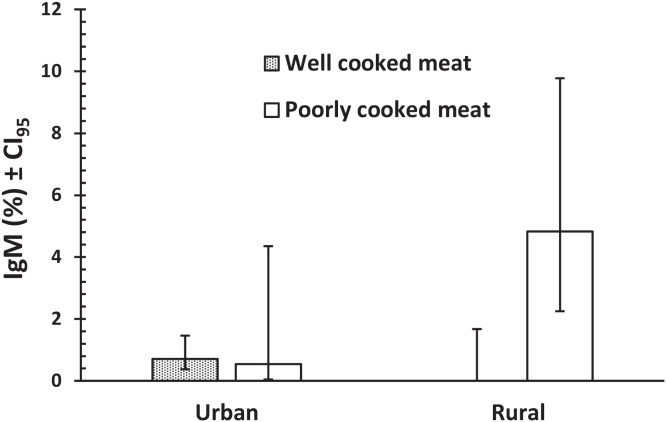
Prevalence of IgM positive samples in urban and rural areas, among subjects who consumed well-cooked or poorly cooked meat.

**Figure 2.**
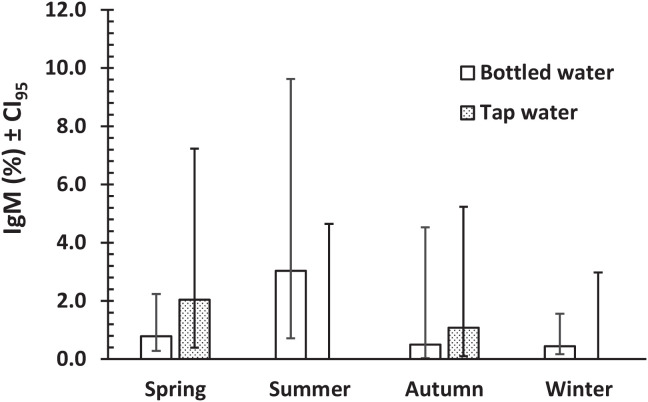
Prevalence of IgM positive samples during four seasons of the year, among subjects who drank bottled or tap water.

Multifactorial analysis in three stages (intrinsic, extrinsic, and behavioral factors, and then factors with *P* <0.3 in first two stages) revealed only one complex interaction in a model comprising only the behavioral factors with IgM (water × meat × garden × IgM; χ^2^_1_ = 4.69, *P =* 0.030), which was not explored further. However, we next fitted a model with water, meat, and garden and also with age, season, and location because the latter three all showed *P*-values of approximately 0.2–0.3. This generated three relevant interactions, the most significant of which was meat × location × IgM (χ^2^_1_ = 12.65, *P* <0.001). This is illustrated in Figure 1, which shows that although IgM prevalence was low among urban dwellers irrespective of whether they consumed well or poorly cooked meat, it was higher among rural dwellers who regularly ate poorly cooked meat. The second interaction comprised season × water × IgM (χ^2^_3_ = 11.41, *P =* 0.010). Figure 2 shows that prevalence of IgM positive samples was higher among those using bottled water in the summer months, when prevalence was zero among those using tap water. In other seasons, there was little difference between those using these two types of water resource. The third interaction was garden × season × IgM (χ^2^_3_ = 7.96, *P =* 0.047), but this was only just significant and was not explored further.

## DISCUSSION

The overall prevalence of anti–*T. gondii* IgG antibodies observed in this study was 13.6%, indicating that these women, with positive IgG titers, has experienced infection in the past and probably had acquired resistance to further infection. However, 86.4% of the pregnant women had no IgG antibodies to the parasite and therefore were susceptible to infection if exposed during the course of pregnancy and, depending on the trimester, were capable of transmitting the infection to the fetus. Therefore, serological monitoring is required during each trimester, with prophylaxis to follow throughout the pregnancy in positive cases.

When we compared our findings with those of previous studies reporting prevalence of toxoplasmosis among pregnant women in different cities in Algeria, we found that *T. gondii* seroprevalence in these other regions, characterized by lower altitudes and with higher humidity, was higher than in the current work (e.g., in Annaba, northeastern Algeria, where prevalence has been reported to be 47.8%^13^ and 52.6%^10^). It is also lower than in Medea, northern Algeria (25%).^14^ This difference could be attributed to the high relative humidity that typifies northern Algeria (center, eastern, and western), which is likely to enhance oocyst survival in the environment preserving viability for longer compared with in the south of Algeria where the climate is much drier. In addition, our results remain lower than those reported in neighboring North African countries, in Europe, and in Asia: 43% in Morocco,^17^ 45.6% in Tunisia,^18^ 50.8% in Libya,^19^ 40.1% in Egypt,^20^ 31.3% in France,^21^ 55.8% in Romania,^22^ 31% in Turkey,^23^ 35.1% in Qatar,^24^ 82.6% in Lebanon,^25^ and 21.2% in Yemen.^5^ However, the results of our survey are similar to those reported in Palestine (17.6%),^26^ Sri Lanka (12.3%),^27^ and Italy (13.8%).^28^ Indeed, variation of *T. gondii* seroprevalence among individuals could be attributed to many factors, including climatic conditions (dry climate, rainfall, temperature, soil type, and altitude).

In our study, a significant association was observed between IgG seroprevalence of *T. gondii* and history of spontaneous abortion, and similar findings have been reported in studies from Yemen^5^ and Egypt.^29^ Many studies in the literature suggest a relationship between *T. gondii* infection and spontaneous abortion.^30^ However, in the present study IgG seroprevalence of *T. gondii* infection was not influenced by age, trimester of pregnancy, place of residence, water consumption, meat consumption, garden work, or by presence of cats in the household. These results are strongly supported by a study carried out in Denmark in which the authors found that up to two-thirds of infections could not be explained by the presence of any specific risk factor.^31^ Nevertheless, this finding contrasts with some previous studies, as, for example, in Medea province, where the risk of toxoplasmosis was significantly associated with increased maternal age.^14^ A similar finding was reported in Qatar.^32^ In Annaba, Algeria, consumption of undercooked meat and contact with cats were reported as significant risk factors for higher *T. gondii* seroprevalence.^13^ Cats have been considered to constitute a high risk factor responsible for transmission of infection to intermediate hosts including humans.^33^ In Italy, a seroprevalence of 19.1% was reported, and the key factors that were shown to increase the risk of infection were consumption of raw meat, contact with cats, consumption of unwashed vegetables or fruit, and contact with soil.^34^

In the present study, 12 (0.89%) cases were seropositive for both IgG and IgM antibodies, which suggests acute infections in these individuals. This finding is lower than those reported from previous studies conducted among pregnant women in Algeria, in which seroprevalence of acute infection for both IgG and IgM antibodies ranged from 4.8% to 43.8%.^10^^,^^13^ However, further confirmation by using seroconversion and/or IgG avidity tests was not carried out in the present study.

Generally, IgM antibody is the first immunoglobulin to be synthesized in response to an immunogenic agent and therefore is an indicator of recent infection, which can help in the determination of acute infection. Usually, detectable IgM appears ∼1 week after exposure to *T. gondii*, and its levels then rise, peaking after 1 to 3 months. This is then followed by a slow decrease over the next 9 months until *T. gondii*–specific IgM is no longer detectable. However, one study reported that IgM antibodies persisted and were detectable for more than 2 years in 27% and 9% of women using the immunosorbent agglutination assay and indirect fluorescent antibody test, respectively.^35^ Therefore, the detection of IgM antibodies is not necessarily a marker of recent infection,^36^ and in such cases, positive IgM test results should be confirmed by additional specific serological tests, including the test for IgG avidity^9^ and IgA and IgE antibody assessment and/or molecular evaluation.^37^ Nevertheless, the advantage of avidity testing is that it ensures that unnecessary spiramycin treatment in pregnant women and unjustified long-term follow-up of fetuses and infants are avoided.^38^

In general, serological screening of pregnant women for *T. gondii* infection is systematically requested by gynecologists in Algeria, who usually recommend treatment with spiramycin until delivery (at a rate of 1 g orally every 8 hours) as soon as a diagnosis of maternal infection is established or strongly suspected. Spiramycin is given to prevent vertical transmission of the parasite to the fetus and is therefore only indicated before the appearance of a fetal infection. If fetal infection is detected, or strongly suspected, the recommended treatment consists of pyrimethamine + sulfonamides + folinic acid, replacing spiramycin.

Univariate analysis of risk factors in the current work showed that the consumption of undercooked meat was a significant risk factor associated with seropositivity of *T. gondii*–specific IgM, which is consistent with studies from Annaba, Algeria,^13^ Egypt,^20^^,^^39^ and Yemen.^5^ However, unlike the present study, other reports from Nigeria,^40^ Saudi Arabia,^41^ and Tunisia^42^ have failed to identify any significant association between consumption of raw and undercooked meat and *T. gondii* seropositivity. Indeed, many Algerian women are active and eat lunch outside their homes; therefore, they may contract toxoplasmosis by ingesting contaminated foodstuffs (sandwiches, cold cuts, pâté, and sausage). It is also relevant that several studies have identified the handling of fresh meat before cooking as a significant risk factor with a high likelihood of accidental contamination by meat-borne stages of *T. gondii*.^5^^,^^43^ Therefore, a health education program to increase women’s knowledge about toxoplasmosis, especially of the sources of infection and modes of transmission, is highly recommended. Preventing infection, notably by avoiding eating raw or undercooked meat, and proper handwashing, particularly after handling or processing raw meat during pregnancy, are all to be encouraged.

Multifactorial analysis of data in the current study enabled identification of more complex interactions between risk factors and *T. gondii* seroprevalence. Our analysis revealed that seropositivity of *T. gondii* IgM among pregnant women who consumed undercooked meat was higher if they came from a rural environment rather than from urban locations. The rural population of Laghouat province is mainly engaged in agricultural activities such as livestock husbandry and breeding (sheep, goats, cattle, horses, camels, and chicken), so the high density of domestic animals in rural areas, as well as the favorable environmental conditions for *T. gondii* oocysts to sporulate, both contribute to the risk of infection. It is also relevant that people from this region are known for their dietary preference of meat consumption and, perhaps not surprisingly given these behavioral characteristics of rural communities in the region, the consumption of undercooked meat leads to a higher prevalence of toxoplasmosis. In a seroepidemiological survey of toxoplasmosis conducted among domestic animals elsewhere in Algeria, the highest prevalence of toxoplasmosis was reported in goats (33.61%), followed by sheep (22.57%) and then cattle (20.04%).^44^ These findings suggest that eating or handling raw infected sheep and goat meat, drinking their raw milk, or tasting any of these during cooking may increase the risk of *Toxoplasma* infection in pregnant women. Also, the preferred habit of eating some traditional Algerian milk products, locally known as Jben cheese, which is made from raw ovine, bovine, and caprine milks and traditional meat meals such as shawarma and brain tissue, may also contribute to and exacerbate the risk of contracting toxoplasmosis in the study population. Further studies to investigate the occurrence of toxoplasmosis in animals in the province are urgently required.

Significantly, increased *T. gondii* seroprevalence was also found among those using bottled water compared with those using tap water in the summer months, and both sources of water in other seasons. Waterborne transmission of *T. gondii* has been reported in different parts of the world, including Yemen,^44^ Nigeria,^45^ Brazil,^46^ and Ethiopia.^47^ Our results may be explicable by the climatic conditions in Laghouat province, which are cooler and drier in winter but warmer and relatively humid in spring and autumn and extremely hot in the summer. Perhaps, stored bottled water provides a more conducive environment for the oocysts of *T. gondii*, enabling them to remain infectious in water for longer periods over the summer months.

We do acknowledge some limitations in our study. First, a cross-sectional study design does not allow inference of causal relationships between *Toxoplasma* infection and the significant risk factors. Second, this study was based on a limited population of pregnant women attending only three private laboratories in Laghouat province, which may not represent the general population in the country. Third, a follow-up serological study of all IgG^−^/IgM^+^ participants was not conducted to confirm seroconversion to IgG and IgG avidity tests were not conducted in these groups of participants.

In the current study, seroprevalence of toxoplasmosis in pregnant women in Algeria was low compared with that in many other studies. Given the presence of *T. gondii* in the local environment and 86.4% seronegativity among pregnant women, the risk of primary acute infection during pregnancy was not high but is nevertheless deserving of attention because of the potentially adverse effects on the fetus. Rural dwellers who frequently ate undercooked meat, women with a history of spontaneous abortion, and consumption of tap water in spring and autumn months or bottled water in the summer were associated with a significantly higher risk of contracting *T. gondii* infection. Antenatal screening of pregnant women and health educational programs about the transmission routes and risk factors for contracting toxoplasmosis in rural areas are strongly recommended.

## References

[1] Tenter AM Heckeroth AR Weiss LM, 2000. *Toxoplasma gondii*: From animals to humans. Int J Parasitol 30: 1217–1258. 11113252 10.1016/s0020-7519(00)00124-7 PMC3109627

[2] Robert-Gangneux F Murat JB Fricker-Hidalgo H Brenier-Pinchart MP Gangneux JP Pelloux H, 2011. The placenta: A main role in congenital toxoplasmosis. Trends Parasitol 27: 530–536. 22079164 10.1016/j.pt.2011.09.005

[3] Ayoade F Todd J Al-Delfi F King J, 2017. Extensive brain masses and cavitary lung lesions associated with toxoplasmosis and acquired immunodeficiency syndrome. Int J STD AIDS 28: 1150–1154. 28632476 10.1177/0956462417696216

[4] Tegegne D Abdurahaman M Mosissa T Yohannes M, 2016. Anti-toxoplasma antibodies prevalence and associated risk factors among HIV patients. Asian Pac J Trop Med 9: 460–464. 27261854 10.1016/j.apjtm.2016.03.034

[5] Al-Adhroey AH Mehrass AAO Al-Shammakh AA Ali AA Akabat MYM Al-Mekhlafi HM, 2019. Prevalence and predictors of *Toxoplasma gondii* infection in pregnant women from Dhamar, Yemen. BMC Infect Dis 19: 1089. 31888517 10.1186/s12879-019-4718-4 PMC6937662

[6] Dubey J, 2004. Toxoplasmosis – A waterborne zoonosis. Vet Parasitol 126: 57–72. 15567579 10.1016/j.vetpar.2004.09.005

[7] Torgerson PR Mastroiacovo P, 2013. Global burden of congenital toxoplasmosis: Systematic review. Bull World Health Organ 91: 501–508. 23825877 10.2471/BLT.12.111732 PMC3699792

[8] Sensini A, 2006. *Toxoplasma gondii* infection in pregnancy: Opportunities and pitfalls of serological diagnosis. Clin Microbiol Infect 12: 504–512. 16700697 10.1111/j.1469-0691.2006.01444.x

[9] Teimouri A Mohtasebi S Kazemirad E Keshavarz H, 2020. Role of *Toxoplasma gondii* IgG avidity testing in discriminating between acute and chronic toxoplasmosis in pregnancy. J Clin Microbiol 58: e00505-20. 32321784 10.1128/JCM.00505-20 PMC7448626

[10] Berredjem H Aouras H Benlaifa M Becheker I Djebar MR, 2017. Contribution of IgG avidity and PCR for the early diagnosis of toxoplasmosis in pregnant women from the north-eastern region of Algeria. Afr Health Sci 17: 647–656. 29085392 10.4314/ahs.v17i3.7 PMC5656190

[11] Torgerson PR Mastroiacovo P, 2013. Global burden of congenital toxoplasmosis: Systematic review. Bull World Health Organ 91: 501–508. 23825877 10.2471/BLT.12.111732 PMC3699792

[12] Peyron F , 2007. Congenital toxoplasmosis in France and the United States: One parasite, two diverging approaches. PLoS Negl Trop Dis 11: e0005222. 10.1371/journal.pntd.0005222 PMC5312802 28207736

[13] Messerer L Bouzbid S Gourbdji E Mansouri R Bachi F, 2014. Séroprévalence de la toxoplasmose chez les femmes enceintes dans la wilaya d’Annaba, Algérie. Rev Epidemiol Sante Publique 62: 160–165. 24661506 10.1016/j.respe.2013.11.072

[14] Khames M Sihem S Hizia H Nguewa P, 2020. High toxoplasmosis seroprevalence among young pregnant women in Medea, Algeria. Ann Parasitol 66: 509–515. 33730475 10.17420/ap6604.292

[15] Rohlf FJ Sokal RR, 1995. *Statistical Tables*. 3rd ed. San Francisco, CA: W.H. Freeman and Company.

[16] Abu-Madi MA Behnke JM Boughattas S Al-Thani A Doiphode SH Deshmukh A, 2016. Helminth infections among long term residents and settled immigrants in Qatar in the decade from 2005 to 2014: Temporal trends and varying prevalence among subjects from different regional origins. Parasit Vectors 9: 153. 26984202 10.1186/s13071-016-1433-5 PMC4793708

[17] Laboudi M Taghy Z Duieb O Peyron F Sadak A, 2021. *Toxoplasma gondii* seroprevalence among pregnant women in Rabat, Morocco. Trop Med Health 49: 21. 33685529 10.1186/s41182-021-00311-5 PMC7941977

[18] Ben Abdallah R Siala E Bouafsoun A Maatoug R Souissi O Aoun K Bouratbine A, 2013. [Toxoplasmosis mother-to-child screening: study of cases followed in the Pasteur Institute of Tunis (2007–2010)]. Bull Soc Pathol Exot 106: 108–112. 23576025 10.1007/s13149-013-0287-8

[19] Mahmoud AS Alarwiy AO Ganghish KS Alhares A Sabei L Altaesh M Algeriany M, 2019. Seroprevalence and potential risk factors associated with *Toxoplasma gondii* infection in women from Tripoli, Libya. Am J Prev Med Public Health 5: 45–49.

[20] Mandour AM Mounib MEM Eldeek HEM Ahmad AAR Abdel Kader AAM, 2017. Prevalence of congenital toxoplasmosis in pregnant women with complicated pregnancy outcomes in Assiut Governorate, Egypt. J Adv Parasitol 4: 1–8.

[21] Robinson E de Valk H Villena I Le Strat Y Tourdjman M, 2021. National perinatal survey demonstrates a decreasing seroprevalence of Toxoplasma gondii infection among pregnant women in France, 1995 to 2016: Impact for screening policy. Euro Surveill 26: 1900710. 33541484 10.2807/1560-7917.ES.2021.26.5.1900710 PMC7863230

[22] Olariu TR Ursoniu S Hotea I Dumitrascu V Anastasiu D Lupu MA, 2020. Seroprevalence and risk factors of Toxoplasma gondii infection in pregnant women from Western Romania. Vector-Born Zoonotic Dis 20: 763–767. 10.1089/vbz.2019.2599 32589521

[23] Tanrıverdi EÇ Kadıoğlu BG Alay H Özkurt Z, 2018. Retrospective evaluation of anti-*Toxoplasma gondii* antibody among first trimester pregnant women admitted to Nenehatun maternity hospital between 2013–2017 in Erzurum. Turkiye Parazitol Derg 42: 101–105. 29780008 10.5152/tpd.2018.5544

[24] Abu-Madi MA Behnke JM Prabhaker KS Al-Ibrahim R Lewis JW, 2010. Intestinal helminthes of feral cat populations from urban and suburban district of Qatar. Vet Parasitol 168: 284–292. 20031329 10.1016/j.vetpar.2009.11.027

[25] Nahouli H El Arnaout N Chalhoub E Anastadiadis E El Hajj H, 2017. Seroprevalence of anti-*Toxoplasma gondii* antibodies among Lebanese pregnant women. Vector Borne Zoonotic Dis 17: 785–790. 29064352 10.1089/vbz.2016.2092

[26] Nijem KI Al-Amleh S, 2009. Seroprevalence and associated risk factors of toxoplasmosis in pregnant women in Hebron district, Palestine. East Mediterr Health J 15: 1278–1284. 20214142

[27] Chandrasena N Herath R Rupasinghe N Samarasinghe B Samaranayake H Kastuririratne A Renuka de Silva N, 2016. Toxoplasmosis awareness, seroprevalence and risk behaviour among pregnant women in the Gampaha district, Sri Lanka. Pathog Glob Health 110: 62–67. 27092763 10.1080/20477724.2016.1173325 PMC4894262

[28] Fanigliulo D Marchi S Montomoli E Trombetta CM, 2020. *Toxoplasma gondii* in women of childbearing age and during pregnancy: Seroprevalence study in central and southern Italy from 2013 to 2017. Parasite 27: 2. 31934847 10.1051/parasite/2019080 PMC6959136

[29] Tammam AE Haridy MAM Abdellah AH Ahmed SR Fayed HM Alsamani MA, 2013. Seroepidemiology of *Toxoplasma gondii* infection in women with first trimester spontaneous miscarriage in Qena Governorate, Egypt. J Clin Diagn Res 7: 2870–2873. 24551661 10.7860/JCDR/2013/6480.3780 PMC3919322

[30] Kalantari N Gorgani-Firouzjaee T Moulana Z Chehrazi M Ghaffari S, 2021. *Toxoplasma gondii* infection and spontaneous abortion: A systematic review and meta-analysis. Microb Pathog 158: 105070. 34186117 10.1016/j.micpath.2021.105070

[31] Petersen E Vesco G Villari S Buffolano W, 2010. What do we know about risk factors for infection in humans with *Toxoplasma gondii* and how can we prevent infections? Zoonoses Public Health 57: 8–17. 19744301 10.1111/j.1863-2378.2009.01278.x

[32] Abu-Madi MA Al-Molawi N Behnke JM, 2008. Seroprevalence and epidemiological correlates of *Toxoplasma gondii* infections among patients referred for hospital-based serological testing in Doha, Qatar. Parasit Vectors 1: 39. 18937857 10.1186/1756-3305-1-39 PMC2586624

[33] Dubey JP, 2009. History of the discovery of the life cycle of *Toxoplasma gondii.* Int J Parasitol 39: 877–882. 19630138 10.1016/j.ijpara.2009.01.005

[34] Thaller R Tammaro F Pentimalli H, 2011. Risk factors for toxoplasmosis in pregnant women in central Italy. Infez Med 19: 241–247. 22212163

[35] Gras L Gilbert RE Wallon M Peyron F Cortina-Borja M, 2004. Duration of the IgM response in women acquiring *Toxoplasma gondii* during pregnancy: Implications for clinical practice and cross-sectional incidence studies. Epidemiol Infect 132: 541–548. 15188723 10.1017/s0950268803001948 PMC2870133

[36] Villard O Cimon B L’Ollivier C Fricker-Hidalgo H Godineau N Houze S Paris L Pelloux H Villena I Candolfi E, 2016. Serological diagnosis of *Toxoplasma gondii* infection: Recommendations from the French national reference center for toxoplasmosis. Diagn Microbiol Infect Dis 84: 22–33. 26458281 10.1016/j.diagmicrobio.2015.09.009

[37] Pomares C Montoya JG, 2016. Laboratory diagnosis of congenital toxoplasmosis. J Clin Microbiol 54: 2448–2454. 27147724 10.1128/JCM.00487-16 PMC5035424

[38] Robert-Gangneux F Dardeá ML, 2012. Epidemiology of and diagnostic strategies for toxoplasmosis. Clin Microbiol Rev 25: 264–296. 22491772 10.1128/CMR.05013-11 PMC3346298

[39] Kamal AM Ahmed AK Abdellatif MZM Tawfik M Hassan EE, 2015. Seropositivity of toxoplasmosis in pregnant women by ELISA at Minia University Hospital, Egypt. Korean J Parasitol 53: 605–610. 26537040 10.3347/kjp.2015.53.5.605 PMC4635823

[40] Deji-Agboola AM Busari OS Osinupebi OA Amoo AOJ, 2011. Seroprevalence of *Toxoplasma gondii* antibodies among pregnant women attending antenatal clinic of Federal Medical Center, Lagos, Nigeria. Int J Biol Med Res 2: 1135–1139.

[41] Almushait MA Bin Dajem SM Elsherbiny NM Eskandar MA Al Azraqi TA Makhlouf LM, 2014. Seroprevalence and risk factors of *Toxoplasma gondii* infection among pregnant women in south western, Saudi Arabia. J Parasit Dis 38: 4–10. 24505169 10.1007/s12639-012-0195-z PMC3909581

[42] Boughattas S Ayari K Sa T Aoun K Bouratbine A, 2014. Survey of the parasite *Toxoplasma gondii* in human consumed ovine meat in Tunis City. PLoS One 9: e85044. 24427300 10.1371/journal.pone.0085044 PMC3888417

[43] Iddawela D Pallegoda Vithana SM Ratnayake C, 2017. Seroprevalence of toxoplasmosis and risk factors of *Toxoplasma gondii* infection among pregnant women in Sri Lanka: A cross sectional study. BMC Public Health 17: 930. 29202747 10.1186/s12889-017-4941-0 PMC5716377

[44] Ouchetati I Ouchene-Khelifi NA Ouchene N Khelifi M Dahmani A Haïf A Zeroual F Benakhla A, 2021. Prevalence of *Toxoplasma gondii* infection among animals in Algeria: A systematic review and meta-analysis. Comp Immunol Microbiol Infect Dis 74: 101603. 33385968 10.1016/j.cimid.2020.101603

[45] Mahdy MA Alareqi LM Abdul-Ghani R Al-Eryani SM Al-Mikhlafy AA Al-Mekhlafi AM Alkarshy F Mahmud R, 2017. Community-based survey of *Toxoplasma gondii* infection among pregnant women in rural areas of Taiz governorate, Yemen: The risk of waterborne transmission. Infect Dis Poverty 6: 26. 28190399 10.1186/s40249-017-0243-0 PMC5304399

[46] Ishaku B Ajogi I Umoh J Lawal I Randawa A, 2009. Seroprevalence and risk factors for *Toxoplasma gondii* infection among antenatal women in Zaria, Nigeria. Res J Med Med Sci 4: 483–488.

[47] de Moura L , 2006. Waterborne toxoplasmosis, Brazil, from field to gene. Emerg Infect Dis 12: 326–329. 16494765 10.3201/eid1202.041115 PMC3373086

